# A call for evidence-based health promotion across the MENA region: From research to impact

**DOI:** 10.34172/hpp.025.45629

**Published:** 2025-12-30

**Authors:** Haidar Nadrian

**Affiliations:** ^1^Associate Editor, Health Promotion Perspectives, Tabriz, Iran; ^2^Department of Health Education and Promotion, Faculty of Health, Tabriz University of Medical Sciences, Tabriz, Iran

 Public health policy and practice in the Middle East and North Africa (MENA) have historically been shaped by political, economic, and crisis-driven priorities. While these factors are undeniably influential, the global transition toward evidence-based public health (EBPH) offers a more accountable, effective, and equitable model for improving population health.^1^ Despite significant advances in EBHP in high-income countries, its systematic application remains sporadic and under-prioritized across much of the MENA region. This editorial highlights this critical gap and advocates for a coordinated regional shift toward evidence-informed health promotion.

 Across MENA countries, health education and promotion (HEHP) research has expanded in volume, yet often remains disconnected from policy impact. Studies tend to cluster in the early phases of the evidence cycle—awareness-raising and descriptive research—with insufficient progression toward evidence synthesis, translation, and implementation. This pattern limits the potential of research to inform effective interventions, optimize resource use, and address persistent public health challenges such as non-communicable diseases, mental health, and health inequities.

 The underuse of evidence-based approaches is not merely an academic concern—it is a systemic failing that diminishes the effectiveness and sustainability of health programs. Without robust evidence synthesis, including systematic reviews and contextually adapted guidelines, health interventions risk being inefficient, culturally misaligned, or duplicative. This know-do gap between research production and practical application undermines health outcomes and public trust.

 We propose that the MENA region adopt and adapt established EBPH frameworks—such as the JBI model of evidence-based healthcare^2^—to guide this transition. Doing so will require:

1. Building Regional Capacity: Strengthening expertise in evidence synthesis, knowledge translation, and implementation science through targeted training, collaborations, and academic programs.^3^
2. Fostering Policy-Academia Partnerships: Creating formal and informal platforms for researchers, policymakers, and practitioners to collaboratively define priorities, share evidence, and co-design interventions.^4^
3. Developing Contextualized Models: Encouraging the adaptation of global evidence to local cultural, religious, and health system realities—ensuring relevance and feasibility.^5^
4. Prioritizing Systematic Reviews: Recognizing synthesis research as the cornerstone of evidence-informed practice and supporting regional repositories of localized evidence.^6^

This shift is both an ethical imperative and a strategic necessity. In a region marked by diverse health systems, resource disparities, and shared public health challenges, leveraging evidence can promote equity, enhance accountability, and maximize the return on health investments.^7^ We call on national health institutes, academic networks, regional bodies such as the WHO Eastern Mediterranean Regional Office, and funding agencies to champion this agenda. Let us move together from isolated studies to integrated evidence ecosystems—where research consistently serves population health, from policy to practice.

## Competing Interests

 Haidar Nadrian is the Associate Editor of *Health Promotion Perspectives*.

## Ethical Approval

 Not applicable.
